# Spatiotemporal differences in and influencing effects of per-capita carbon emissions in China based on population-related factors

**DOI:** 10.1038/s41598-023-47209-2

**Published:** 2023-11-17

**Authors:** Hua Zhang, Yi Li, Jiaxuan Tong

**Affiliations:** 1 School of Statistics, Shanxi University of Finance and Economics, Taiyuan, China; 2 School of Information, Shanxi University of Finance and Economics, Taiyuan, China

**Keywords:** Climate-change mitigation, Climate-change mitigation, Climate-change policy, Governance

## Abstract

Intensive human activities and resource consumption in China have led to increasing carbon emissions, placing enormous pressure on achieving sustainable development goals. Nonetheless, the effects of population-related factors and carbon emissions remain controversial. This study focuses on the spatiotemporal differences in and influencing effects of per-capita carbon emissions using 2010–2019 panel data covering 30 regions in China. Differing from previous studies, population-related factors are employed to classify the 30 regions into 4 classes, and kernel density estimation, σ convergence and spatial econometric models are used to analyse the spatiotemporal differences in and influencing effects of per-capita carbon emissions. The results demonstrate that overall per-capita carbon emissions rose, but there was heterogeneity in the change in per-capita carbon emissions in the 4 classes of regions. The difference in regional per-capita carbon emissions has been widening, but the change rate of the difference stabilized. Overall, per-capita carbon emissions are heavily affected by household size; however, the driving forces behind per-capita carbon emissions in the 4 classes of regions vary. These results suggest that precise and coordinated governance of carbon emissions and reverting to the traditional household structure should be considered to meet the dual carbon goal.

## Introduction

The greenhouse effect poses a severe threat to the world's energy, environmental, and sustainable development, and it is principally blamed on excessive CO_2_ emissions^1^. It has been widely acknowledged that industry is responsible for the majority of carbon emissions. Nevertheless, some academics have maintained that, in some nations, energy consumption driven by human requirements and preferences is the primary cause of carbon emissions, with population factors now making a larger contribution to these emissions than the industrial sector^2,3^. Understanding the relationship between population and carbon emissions is challenging because the impact of population on carbon emissions is widespread. Understood from an economic perspective, people's desires and needs drive production processes that consume energy and thus generate carbon emissions; understood from a social perspective, the characteristics of people's social activities, including their size, distribution, structure, aggregation, and quality, are changing, as are their corresponding needs, which further affect energy consumption and carbon emissions. Hence, the connection among population-related factors and carbon emissions has attracted increasing research interest. As the nation with the largest population, China has the highest energy consumption needs, which has inherently led to an abrupt rise in energy usage and, therefore, significant emissions. Studies have shown that population policies are far less costly than other options developed for low-carbon schemes^4,5^. Initially, the focus was on developing low-carbon energy sources to replace the use of fossil fuels, such as solar, wind, and nuclear power and biofuels. However, the costs of capturing and conserving these energy sources were enormous. To maintain energy and environmental sustainability, people are beginning to focus on energy users themselves. From a global perspective, the population factors of different regions are not uniform, and when analysing carbon emissions at the user source, it is a good decision to take population factors into account. Currently, there is not a wide range of studies analysing the factors of carbon emissions from the population dimension, which directly limits the discussion on population policies. Existing studies focus on the economic dimension^6–8^, and the main contribution of these studies is that they reveal the positive role of carbon emissions in energy sustainability from a spatiotemporal perspective. Therefore, clarifying the spatiotemporal differences in and influencing effects of carbon emissions based on a population perspective to formulate cost-effective carbon emission abatement policies is crucial.

To date, numerous studies that consider carbon emissions have already been conducted, chiefly involving economic^9,10^ and technological advancement^11,12^, population growth^13^ and population-related factors^14,15^.

Regarding economic and technological advancement, for instance, scholars have used different data and methods to demonstrate that economic growth and carbon emissions are positively correlated, while technological advancement yields negative impacts^16–19^.

In regard to the effect of population growth on carbon emissions, studies from various eras have adopted distinct perspectives. Earlier studies proposed that population growth affects atmospheric carbon dioxide levels mainly by increasing fossil fuel consumption and destroying forests^20^, and they suggested that population growth and carbon emissions have a short-run dynamic link^21^. Casey and Galor^22^ used an unbalanced annual panel of cross-country data to establish a variant of the stochastic impacts by regression on population, affluence, and technology (STIRPAT) equation; they utilized fertility to indicate population growth and found that lower fertility could diminish carbon emissions. In contrast, Satterthwaite^23^ emphasized that carbon emissions are rising because of upsurging consumer demand and levels of consumption instead of population growth. Most countries with higher population growth rates produce lower per-capita carbon emissions and consider population growth a trivial predictor of carbon emissions^24^.

In addition, the link between population-related factors and carbon emissions has remained a considerable topic of interest. The STIRPAT model can be employed to quantify the effects of population-related factors on carbon emissions, and the findings revealed that various factors can produce unique effects, both positive and negative^25^. Therefore, more notably, considering population-related factors is necessary when examining carbon emissions based on population. Liddle^26^ summarized evidence indicating that population-related factors such as population size, urbanization rate, population growth, household size, age structure, and population density have an effect on carbon emissions and energy consumption. Similar studies have also included migrant population, population distribution, population quality, population living standards, per-capita gross domestic product (GDP), and industrial energy intensity as population-related factors^15,27,28^. It is clear that population size, urbanization rate, household size, and population ageing are indispensable components when examining how population-related factors affect carbon emissions.

Within different research contexts, the conclusions regarding the impacts of population-related factors on carbon emissions differ. Most importantly, the association between population size and carbon emissions is distinct based on data for different periods and regional studies^29,30^. Studies have asserted that population size has a considerably smaller influence on carbon emissions^2,31^. In contrast to this finding, numerous studies focusing on the same issue have been performed by several researchers, and the reported results indicated that population size is the prime cause of the variation in carbon emissions and generates the greatest effect^32–35^. Investigations into how the urbanization rate affects carbon emissions have also been conducted. According to some studies, growing urbanization could cause a rise in carbon emissions because of the industrial structure, transportation effects^36^, and road energy use^37,38^, but this impact varies by region^39^. For example, the urbanization rate exerts little effect on the carbon emissions in Organisation for Economic Co-operation and Development (OECD) countries^40,41^. However, other researchers have discovered that the urbanization rate and carbon emissions have an inverted U-shaped relationship^42,43^. There are conflicting results regarding how household size affects carbon emissions. Some works have found that the larger the household size is, the higher the carbon emissions^27,44^, while others have provided evidence that carbon emissions are hardly dependent on household size^45,46^. Certain studies have suggested that population ageing contributes to carbon emissions^47^, whereas other studies have proposed that population ageing mitigates carbon emissions^48,49^.

In a study of the spatiotemporal differences in carbon emissions, researchers examined carbon emissions in China's underdeveloped, developing, and advanced regions^50^. Their results recommended that carbon emissions in developing regions are more serious than those in other regions. It is clear that there exists spatiotemporal heterogeneity in carbon emissions. Therefore, studying the spatiotemporal differences in carbon emissions is highly significant for narrowing the governance gap and ensuring balanced development in all regions. However, existing work focuses on the division of the study area according to its geographical conditions. For instance, in China, the carbon emission features of the eastern, middle and western regions have been researched^51,52^. Other study regions in terms of carbon emission features include the Yellow River^53^, Yangtze River^52^, Beijing-Tianjin-Hebei region^54^ and some provinces^25,55^. Among them, in terms of study regions, most investigations have concerned national and provincial strata, mainly based on geographical location, with just a few studies considering subregions based on population-related factors.

Based on the above, the existing studies have contributed professional perspectives into the effects of carbon emissions. Owing to the different research variables, regions, methods, and time intervals, population-related factors affect carbon emissions quite separately. Despite the high uncertainties in the different works, identifying their relationships could elucidate the underlying features of carbon emissions. To date, available studies of spatial carbon emissions have employed geographical division, thus disregarding the effects of population elements. Moreover, few studies have considered the influences of population industrial structure and population quality on carbon emissions.

To overcome the above research deficiencies, we expand on previous studies in two aspects. First, population-related factors serve as clustering variables for regional segmentation; simultaneously, the population industrial structure and population quality are included among population-related factors. Second, the density curve and convergence methods were used to analyse the spatiotemporal heterogeneity, convergence, and influencing effects of per-capita carbon emissions, respectively, providing a basis for policy making.

## Data and methods

### Research framework

**Figure 1:**
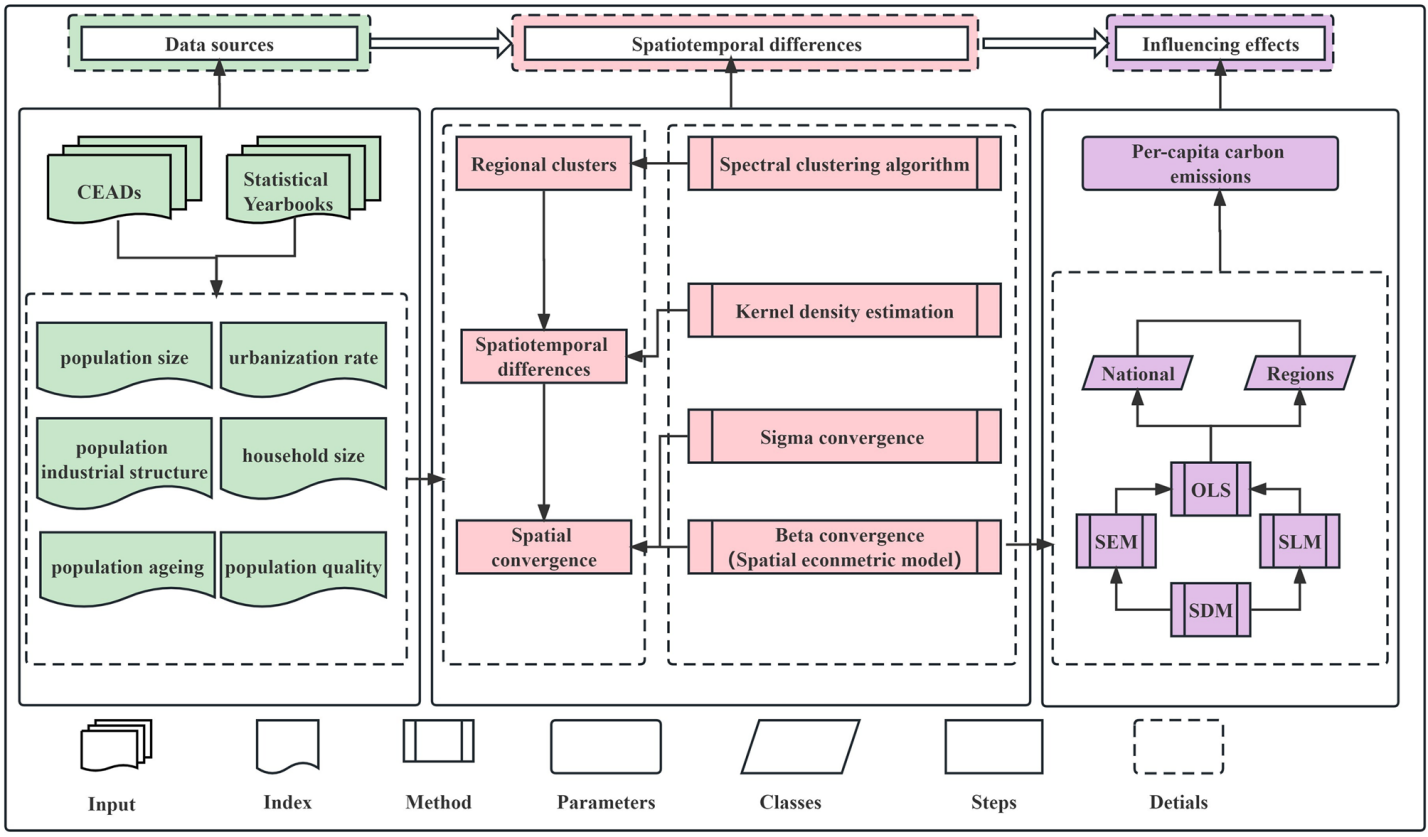
Research framework.

First, we selected population-related indicators that might impact carbon emissions. Second, the 30 study regions were separated into 4 classes using the spectral clustering algorithm based on population-related factors. Subsequently, we revealed the spatiotemporal differences in national carbon emissions considering spatiotemporal evolution and spatial convergence, deploying kernel density estimation, and σ and β convergence based on the per-capita carbon emissions. Finally, spatial econometrics were used to investigate the impacts of population-related factors on per-capita carbon emissions. Figure 1 depicts our research framework.

### Data sources and description

The study area encompasses 30 regions in China. However, Tibet, Hong Kong, Taiwan, and Macao Special are excluded, and the period was set to 2010–2019. The data used for carbon emissions and population-related factors were obtained from the China Emission Accounts and Datasets (CEADs), China statistical yearbooks (2011–2020) and provincial statistical yearbooks (2011–2020).

We disaggregated the population-related factors into six variables: population size, urbanization rate, population industrial structure, household size, population ageing, and population quality. The variables of interest are the per-capita carbon emissions. Table 1 lists the names, units and definitions of all variables.

**Table 1:** Names, units and definitions of all the variables.

Variable	Name	Unit	Definition
*Y*	Per-capita carbon emissions	Tons per person	The ratio of the total carbon emissions to the population size
*X*_*1*_	Population size	Million persons	Total population year ended
*X*_*2*_	Urbanization rate	Percent	The ratio of the permanent resident population living in cities to the population size
*X*_*3*_	Population industrial structure	Percent	The ratio of employed individuals in the secondary industry to all employed individuals
*X*_*4*_	Household size	Persons per household	The number of individuals with consanguinity, marriage, and adoption ties
*X*_*5*_	Population ageing	Percent	The ratio of individuals older than 65 relatives to the population size
*X*_*6*_	Population quality	Percent	The ratio of literate individuals over 15 years

### Research methods

#### Spectral clustering

Spectral clustering evolved from graph theory and is mainly used to cut graphs, but there is no way to cluster discrete clusters^56^, which renders this method more appropriate for the small-sample-situation of the 30 regions in this research. The spectral clustering processes are summarized in Table 2.

**Table 2:** Spectral clustering.

Spectral clustering algorithm
**Input:** Dataset, similarity matrix generation method, reduced dimension k_1,_ clustering method, number k_2_ of clusters
**Output:** Class *C (c*_*1*_*, …., c*_*k2*_*)*
1. The similarity matrix *S* is generated according to the sample points 2. The adjacency matrix *W*, the degree matrix *D*, and the Laplacian matrix* L* are constructed 3. The smallest *k*_*1*_ eigenvalues and the corresponding eigenvector f in 4 are determined 4. The rows of the matrix of *k*_*1*_ vectors are clustered by the clustering method 5. The clustering results class *C (c*_*1*_*, …., c*_*k2*_*)* are obtained

There are two main steps. The first step is composition: we compiled the sampling point data into a network diagram using the fully connected approach method. A similarity matrix was generated by using the similarity distance among the sample points utilizing the Gaussian kernel function. The similarity distance can be computed as follows:

1 $$s_{i,j} = e^{{\frac{{ - \left\| {x_{i} - x_{j} } \right\|^{2} }}{{2\sigma^{2} }}}}$$

where *i* and *j* are two sample points; the similarity matrix is *S*: *S*_*i, j*_ = [*s*]_*i, j*_. The similarity matrix *S* can be further reconstructed into the adjacency matrix *W*. In the Gaussian kernel method, the similarity matrix and adjacency matrix are the same, and these are the definitions for the degree matrix *D*:

2 $$D_{i,j} = \left\{ \begin{gathered} 0,{\kern 1pt} {\kern 1pt} {\kern 1pt} {\kern 1pt} {\kern 1pt} {\kern 1pt} {\kern 1pt} {\kern 1pt} {\kern 1pt} {\kern 1pt} {\kern 1pt} {\kern 1pt} {\kern 1pt} {\kern 1pt} {\kern 1pt} {\kern 1pt} {\kern 1pt} {\kern 1pt} {\kern 1pt} {\kern 1pt} {\kern 1pt} {\kern 1pt} {\kern 1pt} {\kern 1pt} {\kern 1pt} {\kern 1pt} {\kern 1pt} {\kern 1pt} {\kern 1pt} {\kern 1pt} {\kern 1pt} {\kern 1pt} {\kern 1pt} if{\kern 1pt} {\kern 1pt} {\kern 1pt} {\kern 1pt} {\kern 1pt} {\kern 1pt} i \ne j \hfill \\ \sum\nolimits_{j} {w_{i,j} ,{\kern 1pt} {\kern 1pt} {\kern 1pt} {\kern 1pt} {\kern 1pt} {\kern 1pt} {\kern 1pt} {\kern 1pt} if{\kern 1pt} {\kern 1pt} {\kern 1pt} {\kern 1pt} {\kern 1pt} {\kern 1pt} {\kern 1pt} i = j} \hfill \\ \end{gathered} \right.$$

where *w*_*i,j*_ denotes elements in *W* and $\sum\nolimits_{j} {w_{i,j} }$ is the total weight of the edges connecting a point to other points in the graph. In addition, the Laplacian matrix *L* = *D-W* is calculated. The second step is graph cutting. The Ncut method is commonly adopted. Ncut seeks to minimize the total number of edges on the subgraphs

3 $$min{\kern 1pt} {\kern 1pt} {\kern 1pt} {\kern 1pt} {\kern 1pt} {\kern 1pt} {\kern 1pt} {\kern 1pt} Ncut(A_{1} ,A_{2} , \cdots ,A_{k} )$$

where *A*_*1*_*, A*_*2*_*, …, A*_*k*_ is a subset of the set of all sample points, $A_{1} \cup A_{2} \cup \cdots \cup A_{k} = U$ and $A_{1} \cap A_{2} \cap \cdots \cap A_{k} = \emptyset$.

#### Kernel density estimation

Kernel density estimation is a method of nonparametric estimation and is suitable for distribution estimation without prior information. One of the advantages of kernel density estimation over histograms is that it can be used in multidimensional space. The multidimensional kernel density can be estimated as:

4 $$\hat{f}_{h} \left( {x_{1} , \cdots ,x_{d} } \right) = \frac{1}{{nh_{prod} }}\sum\nolimits_{i = 1}^{n} {\prod\nolimits_{j = 1}^{d} {K\left( {\frac{{x_{j} - x_{ij} }}{{h_{j} }}} \right)} }$$

where *h*_*prod*_ = *h*_*i*_ × … × *h*_*d*_,(*h*_*i*_ × … × *h*_*d*_)^*T*^ denotes the bandwidth, *h* is the window width, *k(t)* represents a standard kernel function, *d* is the dimension, and the number of sample points is denoted by *n*. Bandwidth size determines curve smoothness and variance. Considering the image visibility and accuracy, we set *h* = 0.15. Multidimensional kernel density estimation enables verification of spatiotemporal differences in per-capita carbon emissions across regions.

#### Convergence of σ and β

We examined the changing trend in China's per-capita carbon emissions differences via two approaches: σ and β convergence. In regard to σ convergence, we determined whether there exists a trend towards convergence in regional differences in per-capita carbon emissions, while β convergence was considered to determine whether the changes in per-capita carbon emissions converge to the same steady state.

(1) Convergence of σ

The economic implication of σ convergence is that the dispersion of the per-capita output among different economies gradually decreases over time^57^. Moreover, σ convergence of per-capita carbon emissions indicates that the difference in per-capita carbon emission level between regions gradually decreases over time, and eventually all regions reach the same level. We utilized the coefficient of variation method, which can be expressed as:

5 $$\sigma_{j} = \frac{{\sqrt {\sum\nolimits_{i}^{{N_{j} }} {(D_{ij} - \overline{D}_{j} )^{2} /N_{j} } } }}{{\overline{D}_{j} }}$$

where *D*_*ij*_ denotes the per-capita carbon emissions in region *i* of class *j*, and the average per-capita carbon emissions in class *j* are indicated by $\overline{D}_{j}$.

(2) Convergence of β

In regard to β convergence, we mainly determined whether there exists a convergence trend in regional development from a growth rate perspective, which can be classified as absolute and conditional β convergence. Among them, absolute β convergence emphasizes the convergence state of per-capita carbon emission development, while conditional β convergence represents the convergence trend of per-capita carbon emission development between regions after governing for numerous influencing factors.

The degree of geographical dependence between regions increases with population mobility. As a result, a β convergence study based on the spatial econometric model may be adopted when there exists a substantial spatial correlation between local intervals. Based on panel data, the absolute β convergence model is:

6 $$\ln \left( {\frac{{y_{i,t + 1} }}{{y_{i,t} }}} \right) = \alpha + \beta \ln \left( {y_{i,t} } \right) + \rho \sum\nolimits_{j = 1}^{N} {w_{ij} } \ln \left( {\frac{{y_{j,t + 1} }}{{y_{j,t} }}} \right) + \theta \sum\nolimits_{j = 1}^{N} {w_{ij} ln\left( {y_{j,t} } \right)} + u_{i} + v_{i} + \varepsilon_{it} , \, \varepsilon_{it} = \lambda \sum\nolimits_{j = 1}^{N} {w_{ij} } \varepsilon_{jt} + \sigma_{it}$$

where *i* denotes each region, *y* is the variable to be studied, *t* is the study time, *β* represents the convergence rate, and if *β* < 0 and the significance test is passed, this indicates that there is a convergence trend in per-person carbon emission levels. The rate of convergence is *v* = *-ln(1* + *β)/T* (T denotes the study period). Moreover, *α* generalizes unobserved parameters such as the steady state, and *u*_*i*_ and *v*_*t*_ denote the spatial fixed effect and temporal fixed effect, respectively. $\varepsilon_{i,t}$ denotes random interference terms that are independent and identically distributed, λ is the spatial error coefficient, *ρ* refers to the spatial lag coefficient, $\theta$ indicates the effect of the spatial lag value of the base period index on the explained variables, and *w*_*ij*_ is the spatial weight matrix's column *j* element in row *i*. For *λ* = 0, we obtained the spatial Durbin model (SDM), for λ = 0 and $\theta$ = 0, we obtained the spatial lag model (SLM), and for λ = 0 and *ρ*-$\theta$ *β* = 0, we obtained the spatial error model (SEM); if the above judgement conditions are true, the ordinary least square (OLS) model applies^58^.

Similarly, based on panel data, the expression of the conditional β convergence model is:

7 $$\begin{aligned} \ln \left( {\frac{{y_{i,t + 1} }}{{y_{i,t} }}} \right) & = \alpha + \beta \ln \left( {y_{i,t} } \right) + \rho \sum\nolimits_{j = 1}^{N} {w_{ij} } \ln \left( {\frac{{y_{j,t + 1} }}{{y_{j,t} }}} \right) + \gamma \ln X_{i,t + 1} + \theta_{0} \sum\nolimits_{j = 1}^{N} {w_{ij} ln\left( {y_{j,t} } \right)} \\ &\quad+ \theta \sum\nolimits_{j = 1}^{N} {w_{ij} ln\left( {X_{j,t} } \right)} + u_{i} + v_{i} + \varepsilon_{it} , \, \hfill \\&\quad \varepsilon_{it} = \lambda \sum\nolimits_{j = 1}^{N} {w_{ij} } \varepsilon_{jt} + \sigma_{it} \hfill \\ \end{aligned}$$

where *X* is the set of population-related factors. In addition, conditional β convergence was utilized to learn the key population-related factors affecting per-capita carbon emissions in each region. *w* is the spatial geographical distance matrix, which is measured by latitude and longitude. The software is ArcGIS 10.8.

## Results

### Regional division based on population-related factors

**Figure 2:**
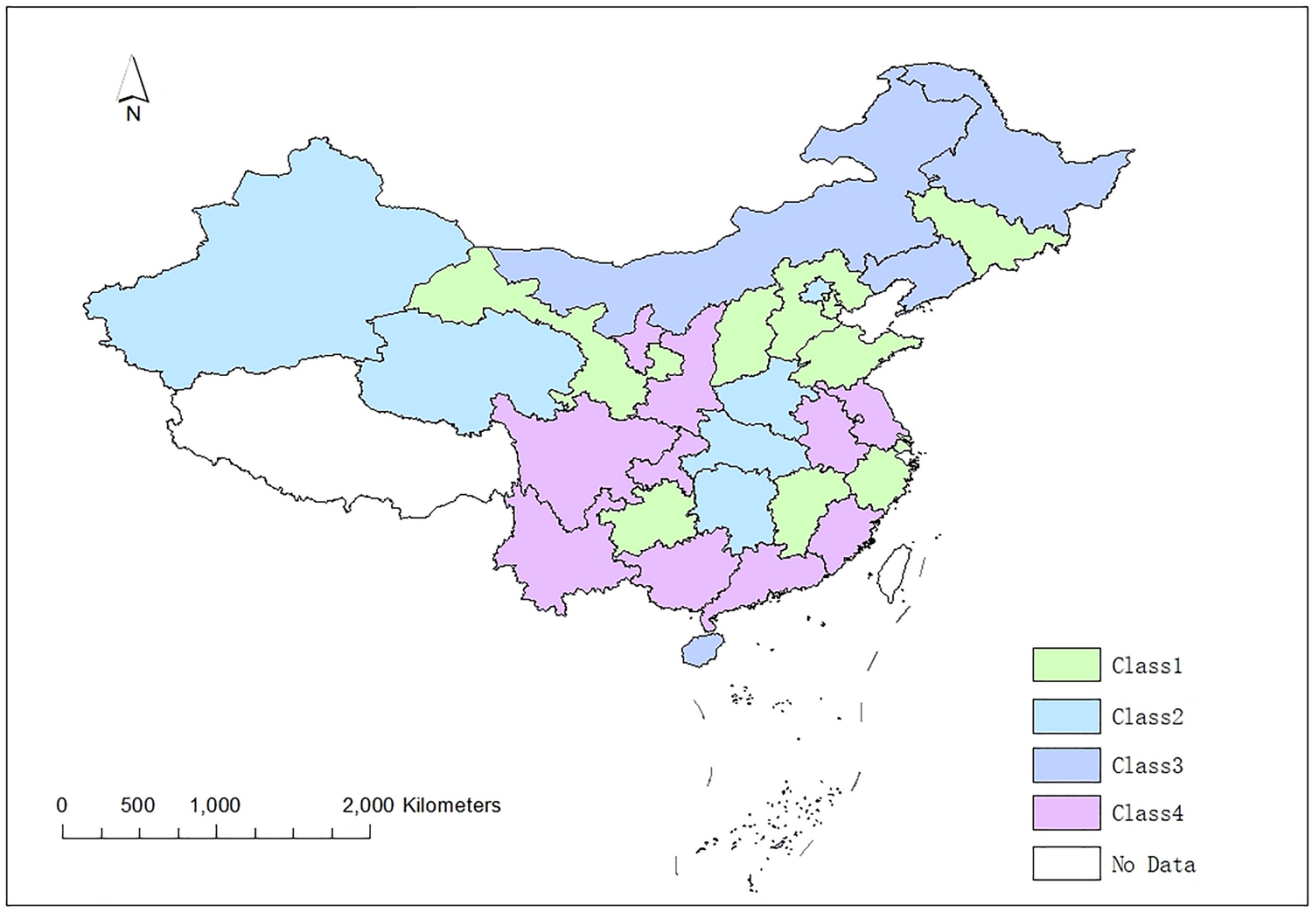
Map of the spectral clustering results.

The mean of the indicators of the six population-related factors over the ten-year period from 2010 to 2019 served to classify the 30 regions of China into 4 classes, thereby utilizing MATLAB 2016 software. In this section, the continuous-type variables were transformed into categorical variables. If the index value remains within 33.33% of the overall data, it occurs at a low level and is recorded as 1; if the index value varies between 33.33 and 66.66% of the overall data, it occurs at the medium level and is recorded as 2; if the index value is above 66.66% of the overall data, it occurs at a high level and is recorded as 3. We mapped the clustering results in geographic information system (GIS) software to display the spatial features of the clustering results more intuitively. Table 3 and Fig. 2 show the outcomes. From Fig. 2, these regional clustering results are distinguished from those obtained in other studies. These four classes of regions are spatially linked but not entirely contiguous. Next, the spatiotemporal differences and spatial convergence of each class and nation were carefully analysed.

**Table 3:** Spectral clustering results.

Class	Regions
Class 1	Tianjin, Hebei, Shanxi, Jilin, Shanghai, Zhejiang, Jiangxi, Shandong, Guizhou, Gansu
Class 2	Beijing, Henan, Hubei, Hunan, Qinghai, Xinjiang
Class 3	Liaoning, Hainan, Inner Mongolia, Heilongjiang
Class 4	Jiangsu, Anhui, Fujian, Guangdong, Guangxi, Sichuan, Chongqing, Shaanxi, Yunnan, Ningxia

### Spatiotemporal differences in per-capita carbon emissions

**Figure 3:**
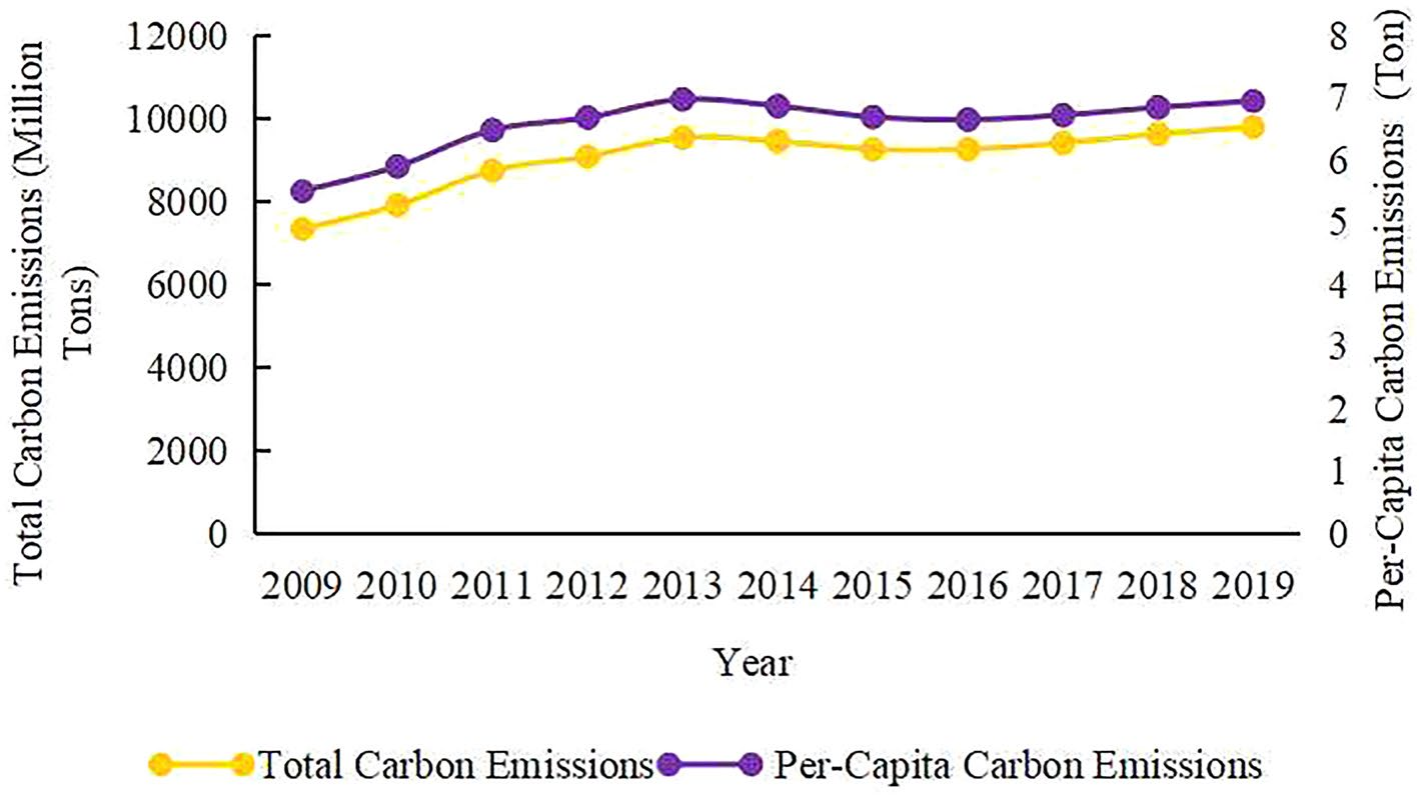
Trends of carbon emissions in China.

As illustrated in Fig. 3, China's total carbon emissions increased from 7333.68 million tonnes in 2009 to 9497.76 million tonnes in 2019. In addition, Fig. 3 displays the change curve of the per-capita carbon emissions in China, in which the shift trend matched that of the total carbon emissions. Thus, we directly selected the index of per-capita carbon emissions for research to ensure a close relationship between the population-related factors and carbon emission analysis.

**Figure 4:**
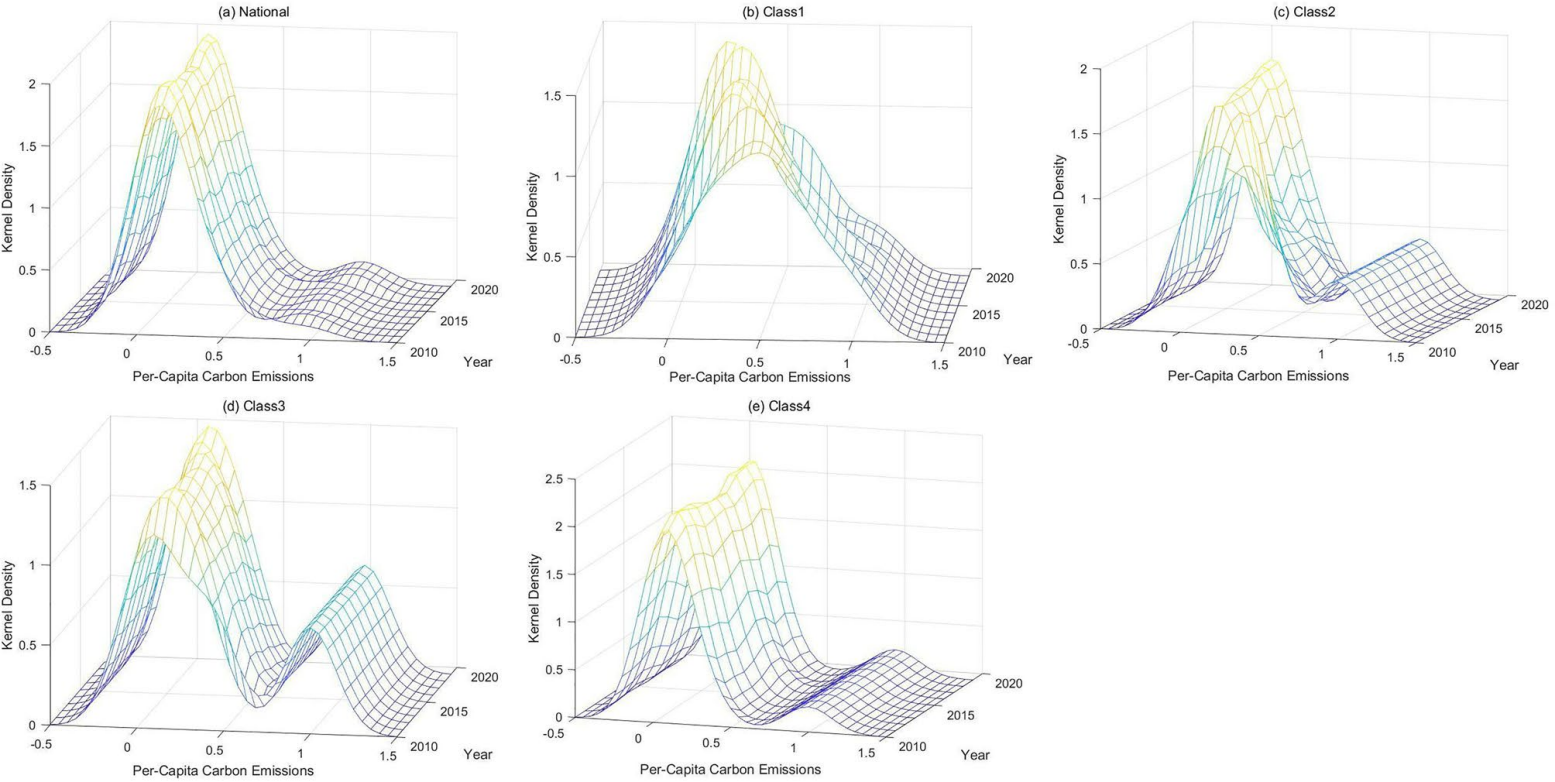
Kernel density estimation curves of the national and four classes of China.

Figure 4 intuitively shows the shifts in China and its 4 classes of regions' per-capita carbon emissions over the three observed years. The distribution location, distribution pattern, and crest number were utilized to summarize the properties of the per-capita carbon emissions. Summarizing this information, we could derive the spatiotemporal differences in per-capita carbon emissions across regions.

(1) Distribution location

The distribution location reflects the extent of per-capita carbon emissions. From Fig. 4, during the observation period, the centre of the distribution curve in the national, class 3 and class 4 regions shifted to the right. This phenomenon shows that the per-capita carbon emission extent nationwide and in the above two classes of regions increased. The distribution curves of the class 1 and 2 regions displayed an obvious leftward shift, suggesting a clear downwards trend in per-capita carbon emissions for these two classes.

(2) Distribution pattern

The spatiotemporal difference in per-capita carbon emissions is reflected in the distribution pattern. The height of the kernel density curve of China's per-capita carbon emissions first decreased and then increased, and the overall performance showed that the height rose and the width widened. These results indicate that China's per-capita carbon emissions experienced a trend of discrete changes and that the differences in per-capita carbon emissions among regions have widened. The height of the kernel density curve of the regions in class 1 generally showed an increasing trend, and the width widened. These results indicate that the internal dispersion increased and that the difference in per-capita carbon emissions in this class of regions expanded. By the same token, the class 2 regional change is as follows: the height first fell and then rose, and the width increased. These results show that the dispersion of indicators within such regions is relatively high. The class 3 regions are as follows: the height and width were similar to the national situation, indicating that the dispersion degree of per-capita carbon emissions increased gradually. The variation in the kernel density curve of the class 4 regions was similar to that of China as a whole and the class 3 regions. These results indicate that the dispersion of per-capita carbon emissions deepened.

Importantly, the divergence trend indicated by the kernel density curve indicates that the difference in per-capita carbon emissions between regions is expanding. In summary, the difference in per-capita carbon emissions is expanding nationwide and within various regions, but the specific time nodes of change are not the same, which demonstrates that the rate of change of per-capita carbon emissions in various classes of regions is different.

(3) Crest Number

The crest number indicates the polarization of per-capita carbon emissions. A bimodal phenomenon was observed for the national class and all classes of regions. This denotes that the development of per-capita carbon emissions exhibited a certain polarization phenomenon and a fixed development difference. The curve of the regions in class 1 showed a unimodal peak at the early stage and a bimodal peak at the late stage, which indicates that the regional differences within the class gradually emerged. The kernel density curves of the emissions in classes 2, 3, and 4 all included a main peak and a lateral peak, which suggests that obvious polarization occurred in these regions.

### Spatial convergence analysis

#### Analysis of σ convergence of per-capita carbon emissions

**Figure 5:**
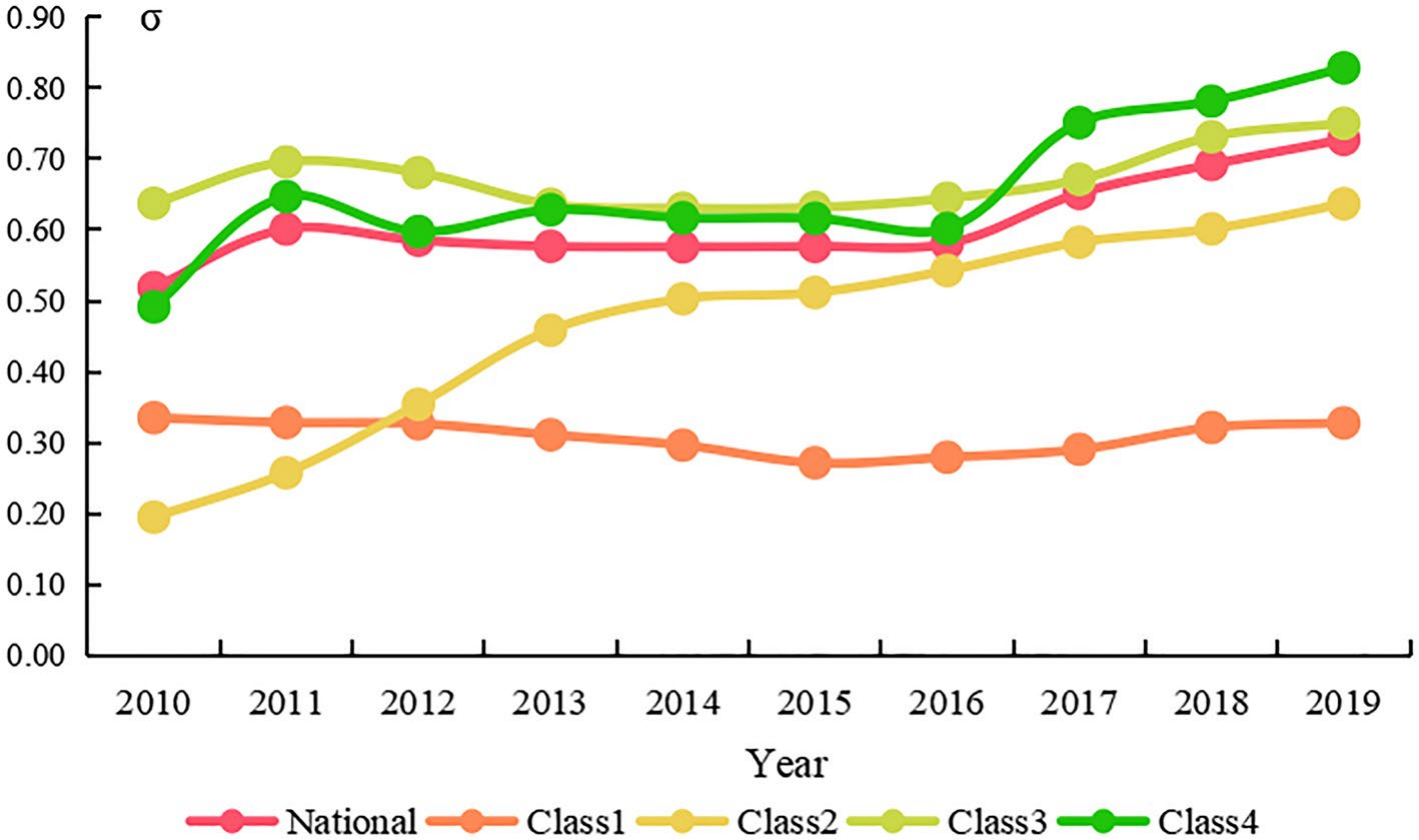
Value of σ convergence of the national and classification regions of China from 2010 to 2019.

Carbon emission convergence is an important prerequisite for China to achieve a carbon peak^59^. As shown in Fig. 5, the available data suggested that there was no significant trend in the diminishment of the regional differences in per-capita carbon emissions. In the national and four regional classes, the trajectory of the emissions showed a considerable increase after 2016. Prior to 2016, the consistency curve of the national σ convergence value remained relatively stable. It was shown that, overall, the regional disparity in emissions was not narrowing but widening. The σ value curves of the class 1 and 3 regions fluctuated, displaying a weak negative trend at first, followed by an increasing trend, which indicates that the difference in the emissions between these two classes of regions decreased at the early stage and that the regional difference returned to an increasing trend until 2015. The σ convergence curve of the class 2 regions indicated a clear increasing trend, which suggests that the regional differences in this class exhibited a notable increasing trend. The curve change of the class 4 regions was tortuous, and there was a relatively large increase after 2016.

#### Analysis of β convergence of the per-capita carbon emissions

Spatial autocorrelation was assessed using Moran’s I before confirming *β* convergence. The computations were completed utilizing Stata 17. The results of Moran’s I are shown in Table 4.

**Table 4:** Moran's I of national per-capita carbon emissions.

Variables	I	P value	Variables	I	P value
D_2010_	0.213	0.002***	D2015	0.160	0.011***
D_2011_	0.156	0.011***	D2016	0.158	0.013***
D_2012_	0.166	0.008***	D2017	0.132	0.024***
D_2013_	0.171	0.008***	D2018	0.143	0.017***
D_2014_	0.169	0.009***	D2019	0.127	0.027***

*P < 0.1, **P < 0.05, and ***P < 0.01.

From Table 4, we see that the national Moran's I of per-capita carbon emissions was constantly positive for 10 years, ranging from 0.127 to 0.213, and all values passed the significance test. Therefore, it can be preliminarily judged that there is spatial autocorrelation in national per-capita carbon emissions.

(1) Estimation model identification

The LM test, Hausman test, fixed effects test, etc., were used to identify the optimal models. We first investigated making the model a more extended SDM and running the LM test to see if there was any spatial connection in the data. If not, we could use the OLS model directly. In the presence of spatial correlation, to evaluate whether the SDM might be degenerated into the SEM or SLM, the Wald test was applied; when all key parameters failed the significance test, the base OLS model was used. To determine whether to utilize random or fixed effects, apply the Hausman test. For example, without considering the influences of population-related variables, the national per-capita carbon emission SDM failed the robustness test (Wald test), demoting the SDM to the SEM or spatial autoregressive (SAR) model, but the significance test was rejected for both the spatial lag coefficient ρ, which is − 0.149, and the spatial error coefficient λ, which is − 0.138. We were restricted to returning to the OLS model as a result, with the Hausman statistic (99.05, P = 0.000) passing the significance test. The final model for absolute β convergence was the fixed OLS model. Displayed in Table 5, in the same way, according to the above analysis steps, the optimal models of classes 1, 2, 3 and 4 were all fixed OLS models. Following consideration of the population-related factors (Table. 6), conditional β convergence of the overall per-capita carbon emissions was further examined. The validation step of the optimal model was consistent with absolute beta convergence analysis, and the optimal models of the national, class 1, 2, 3 and 4 regions were all fixed OLS models.

**Table 5:** Absolute β convergence test results of per-capita carbon emissions.

Regions	National	Class 1	Class 2	Class 3	Class 4
Model type	OLS	OLS	OLS	OLS	OLS
β	− 0.390*** (− 10.37)	− 0.399*** (− 4.50)	− 0.264*** (− 5.750)	− 0.577*** (− 5.19)	− 0.45*** (− 5.98)
α	0.788*** (− 10.65)	0.844*** (− 4.60)	− 1.88 (2.717)	1.285*** (5.32)	0.846 *** (6.14)
R^2^	0.3102	0.204	0.4681	0.465	0.312
LM spatial lag	32.421***	1.133	4.299**	1.305	7.459***
Robust LM spatial lag	5.490**	0.028	0.018	1.944**	0.113
LM spatial error	34.171***	1.11	4.281**	1.308	7.674***
Robust LM spatial error	7.239***	1.252	0.001	1.948*	0.328
Hausman test	99.05***	14.96***	37.58***	9.69***	29.28***

*P < 0.1, **P < 0.05, and ***P < 0.01. The statistic is given in parentheses.

**Table 6:** Conditional β convergence test results of per-capita carbon emissions.

Regions	National	Class 1	Class 2	Class 3	Class 4
Model type	OLS	OLS	OLS	OLS	OLS
β	− 0.382*** (− 8.77)	− 0.428*** (− 3.65)	− 0.3177*** (− 4.74)	− 0.685*** (− 4.38)	− 0.462*** (5.32)
α	2.350*** (− 0.376)	4.166 (0.89)	− 1.88 (− 0.35)	− 6.35 (− 0.65)	− 1.091 (− 0.17)
R^2^	0.340	0.270	0.580	0.681	0.375
lnX_1_	0.104 (− 0.42)	− 0.817* (− 1.96)	0.8364 (1.39)	0.787 (0.59)	0.387 (0.64)
lnX_2_	− 0.050 (− 0.61)	0.288* (1.92)	− 0.357*** (− 2.18)	0.161 (0.26)	− 0.114 (− 0.58)
lnX_3_	− 0.130 (− 0.29)	− 0.151* (− 1.88)	0.117 (0.83)	0.341* (1.74)	0.001 (0.01)
lnX_4_	− 0.320*** (− 2.70)	0.085 (0.34)	0.178 (0.64)	− 1.13*** (− 3)	− 0.42** (− 2.18)
lnX_5_	− 0.009 (− 1.57)	0.009 (0.88)	− 0.25** (− 2.49)	− 0.021* (− 1.74)	− 0.01 (− 0.87)
lnX_6_	− 0.397	0.589 (0.63)	− 0.734 (− 1.1)	0.302 (0.15)	− 0.08 (− 0.08)
LM spatial lag	19.462***	1.139	1.224	0.003	4.508**
Robust LM spatial lag	4.678**	2.645	4.747	0.106	3.847**
LM spatial error	15.921***	0.891	0.143	0.021	3.010*
Robust LM spatial error	1.137	2.397	3.646	0.124	2.348
Hausman test	141***	21.01***	17.11***	7.63***	29.02***

*P < 0.1, **P < 0.05, and ***P < 0.01. The statistic is given in parentheses.

(2) Convergence analysis of the change rate of the regional differences

The regression findings showed that the absolute and conditional convergence coefficient β values at the national level and of classes 1, 2, 3 and 4 were negative, and the significance test was successful, indicating that there occurred absolute and conditional *β* convergence of the per-capita carbon emissions. On a national level, the absolute and conditional convergence rates were 4.932% and 4.813%, respectively. The national per-capita carbon emissions convergence rate, which considers population-related factors, declined relative to the absolute convergence rate, indicating that population-related factors could, by degrees, impede the development of per-capita carbon emissions convergence.

(3) Effects of population-related factors on per-capita carbon emissions

On a national scale, we focused on household size (*X*_*4*_) among the control factors. The regression coefficient of household size was found to be considerably negative, and the significance test was successful, demonstrating that increasing household size had a significant inhibitory influence on per-capita carbon emissions. The effect of the various variables on per-capita carbon emissions revealed regional variances according to the analysis of the different regions. Specifically, in the class 1 regions, the population size, urbanization rate and population industrial structure regression coefficients were − 0.817, 0.288 and − 0.151, respectively, and they were all significant. These results indicate that population size and the population industrial structure positively affect reducing per-capita carbon emissions, but the urbanization rate does not benefit reducing per-capita carbon emissions. These regions focus their governance on the level of urbanization. Within the class 2 regions, the regression coefficients of the urbanization rate and ageing were − 0.357 and − 0.25, respectively, and they were all significant, showing that the urbanization rate and ageing in these regions were beneficial for reducing per-capita carbon emissions. To ensure that these regions reach the stable state faster, it is essential to further coordinate the balanced development of the regional urbanization rate and the population structure as soon as possible. In the class 3 regions, the regression coefficients of the population industrial structure, family size and ageing were 0.341, −1.13 and − 0.021, respectively, and they are all significant, indicating that the population industrial structure in this type of region was unreasonable and could not facilitate the reduction in per-capita carbon emissions, while the household size and ageing could further reduce the emissions. Within the class 4 regions, the factors affecting the per-capita carbon emissions were consistent with those at the national level, and an increase in household size could effectively reduce the per-capita carbon emissions.

## Discussion

### Comparison to previous studies

Scholars' perspectives are polarizing as studies on the influence of population-related factors on carbon emissions become more specialized. Existing research reveals that, depending on the situation, population-related factors have various effects on carbon emissions. We examined the spatiotemporal differences and influencing effects of per-capita carbon emissions against the backdrop of the time before the dual carbon target was proposed.

In contrast to previous similar studies that directly used geographical location to classify regions^60,61^, this paper classifies regions based on the level of population-related factors. Since the spatial distribution of population-related factors is not uniform and continuous, the geographical distribution of regions is spatially linked but not entirely contiguous. In this work, 30 regions in China were divided into 4 classes using clustering characteristics linked to population. The traditional way of dividing regions according to physical geography was abandoned. This regional research method could better highlight the influences of population-related factors on carbon emissions and ensure more realistic research results. Meanwhile, the interval difference of per-capita carbon emissions diverged, indicating that the changes in emissions may be sensitive to the region. Hence, studying the differences in per-capita carbon emissions by region is essential.

Multidimensional kernel density analysis could be utilized to assess the spatiotemporal differences in per-capita carbon emissions from multiple perspectives. The distribution location, pattern and crest number of the curve can intuitively and vividly reveal the trend and polarization phenomenon, which is more specific than the general descriptive statistical analysis method and can reveal deep-level features. The study of σ convergence can help understand the spatiotemporal difference trend of per-capita carbon emissions. We can see that overall, the regional differences in the per-capita carbon emissions are expanding. The study of the spatiotemporal differences in China's per-capita carbon emissions in this paper is in line with the conclusions of similar studies^62^.

Spatial econometric methods can not only be utilized to further analyse the convergence of differences but also be utilized to reveal the factors behind the differences in per-capita carbon emissions. Our findings demonstrated that the changes rates of the regional differences gradually converged to the same steady state. This conclusion is consistent with the results of a similar study^59^, but population-related factors may negatively impact the convergence rate. The factors influencing per-capita carbon emissions and the direction of influence varied by region. The reason is that this paper starts with regional clustering and divides the country into 4 classes using the index level of population-related factors, and the population index level of regions within the classes is similar. The influencing factors of per-capita carbon emissions derived from the analysis of this model are different from those of traditional studies.

### Suggestions for carbon abatement measures

#### Act according to local conditions

The kernel density analysis outputs indicated that the national per-capita carbon emissions curve moved to the right, indicating a rise in these emissions. The distribution locations of the kernel density curves of the 4 classes of regions were not consistent, indicating the existence of spatiotemporal heterogeneity.

The important of the dual carbon target should be acknowledged. In light of the spatiotemporal heterogeneity of per-capita carbon emissions across regions, the government should avoid one-size-fits-all policy formulation and give more attention to differentiated regional carbon emission control measures. The σ convergence analysis results showed that the difference in the per-capita carbon emissions among regions was increasing, and this difference will not disappear automatically. The spatial econometric findings also indicated that the factors influencing per-capita carbon emissions varied from region to region. This also demonstrates the ineffectiveness of one-size-fits-all policies. In other words, when formulating emission reduction policies, the leading role of the central government should be given full play, and local governments should formulate effective and reasonable policies in stages according to their specific per-capita carbon emission conditions. While implementing targeted and accurate governance, we should avoid totally copying already successful governance situations.

The model results show that the existing population size and the proportion of the secondary industry population in class 1 regions contribute to reducing per-capita carbon emissions, while the increase in the urbanization rate may increase carbon emissions. Therefore, carbon emission reduction policies in such regions should focus more on how to achieve urbanization. For class 2 regions, the existing urbanization rate and ageing situation are conducive to reducing per-capita carbon emissions, and such regions should pay attention to the smooth transition of the urbanization rate and ageing while developing. The existing family size and ageing level in class 3 regions can promote a reduction in per-capita carbon emissions, while the increase in the proportion of the population in the secondary industry is not conducive to reducing carbon emissions in such regions. Thus, the development of knowledge-intensive industries should be encouraged in such regions. In class 4 regions, the existing household size helps to reduce per-capita carbon emissions. Therefore, it is recommended that these regions vigorously promote traditional culture and promote family integration.

#### Revert the traditional household structure and promote multigenerational cohabitation.

The β convergence analysis results revealed that the change rate of per-capita carbon emissions will successively converge to the same level. The convergence rate, considering the influence of population-related variables, decreased relative to the absolute β convergence rate, indicating that population-related factors could reduce convergence. The regression model's significance coefficient also demonstrated that household size had a substantial detrimental influence on per-capita carbon emissions.

Based on the aforementioned findings, the regional inequality in carbon emissions per person is expected to aggravate rather than diminish in the upcoming years. As a result, government engagement in carbon emissions governance is essential. In light of the effects of population elements on per-capita carbon emissions, we advocate reverting to the traditional household structure and a large family. Previous research has noted that the decline in household size and the increase in the number of households will cause a rise in the demand for durable consumer items and basic household essentials, boosting carbon emissions^25^, and a similar finding was obtained in this investigation. We can therefore start by expanding household size and formulate policy proposals to indirectly reduce per-capita carbon emissions. The precise strategies are as follows: first, family education and family culture should be improved; family virtues should be promoted; and a correct family concept should be established. The core of family integration is the traditional Chinese notion of the family, which promotes a sense of support for the young and a sense of dependence for the old, and the development of sound family values will aid in increasing household size. Second, favourable family tax policies, such as tax breaks for households with adults 60 and older, should be implemented. On the one hand, they might encourage larger families, and on the other hand, they might ease the burden of caring for older family members on offspring. Third, the comfort and atmosphere of communal living should be enhanced. Care facilities for older individuals should be improved; for instance, older individuals can be jointly housed, which can provide an opportunity to bring together elderly people with no family to form a prominent family of elderly people, while youth housing can achieve the same for young people.

## Conclusions

Based on panel data collected between 2010 and 2019 from 30 Chinese provinces and cities, we used the spectral clustering technique to divide geographical regions and analysed the spatiotemporal heterogeneity and differences in the per-capita carbon emissions through kernel density estimations and the σ convergence model. Finally, the drivers of the progress of per-capita carbon emissions were modelled using the spatial econometric model. The key findings can be summarized as follows:

1. Carbon emissions in China as a whole are increasing. There is spatiotemporal heterogeneity in the per-capita carbon emissions, as evidenced by the distinct change trends of the density curves of the per-capita carbon emissions in the various regions. The dispersion degree of the emissions in the different regions differs, and a polarization phenomenon is observed.
2. The existing difference in per-capita carbon emissions among regions is expanding, but the change rate of the difference is gradually converging to the same level over time, and the impacts of population-related factors can reduce the convergence rate.
3. There is evidence that household size imposes a substantial passive effect on national per-capita carbon emissions. Different classes of regions have various motivating elements for per-capita carbon emissions.

## Data Availability

The datasets used and/or analysed during the current study are available from the corresponding author upon reasonable request.
