# Author Correction: Spatiotemporal differences in and influencing effects of per-capita carbon emissions in China based on population-related factors

**DOI:** 10.1038/s41598-023-48296-x

**Published:** 2023-11-28

**Authors:** Hua Zhang, Yi Li, Jiaxuan Tong

**Affiliations:** 1 School of Statistics, Shanxi University of Finance and Economics, Taiyuan, China; 2 School of Information, Shanxi University of Finance and Economics, Taiyuan, China

Correction to: *Scientific Reports* 10.1038/s41598-023-47209-2, published online 17 November 2023

The Funding section in the original version of this Article was omitted.

The Funding section now reads: The Funding section in the original version of this Article was omitted. The Funding section now reads: “This work was supported in part by the Scientific Activities of Selected Returned Overseas Professionals in Shanxi Province under Grant 20220025, in part by the National Statistical Science Research Project of China under Grant 2022LZ14, in part by the Humanities and Social Sciences Foundation of Ministry of Education, China, under Grant 20YJA910004, and in part by the Shanxi Province Youth Talent Support Program under Grant 20190705.”

The original Article has been corrected.

